# 569 Burn Registries State of Affairs: A Scoping Review

**DOI:** 10.1093/jbcr/irac012.197

**Published:** 2022-03-23

**Authors:** Eduardo I Gus, Stephanie Brooks, Iqbal Multani, Jane Zhu, Jennifer Zuccaro, Yvonne Singer

**Affiliations:** The Hospital for Sick Children, University of Toronto, Toronto, Ontario; University of Toronto, Toronto, Ontario; Alfred Health, Melbourne, Victoria; University of Toronto, Mississauga, Ontario; SickKids, Toronto, Ontario; Victorian Adult Burn Service, The Alfred, Melbourne, Victoria

## Abstract

**Introduction:**

Registry science applies observational study designs to interpret large secondary databases. It can be utilized to understand disease and injury, answer research questions, inform regulatory decision making, and engender benchmarking of quality-of-care indicators. Numerous burn registries exist globally, however their contributions to the science of burn epidemiology, care and treatment have not been summarized. The objective of this study is to characterize the available literature on burn registries.

**Methods:**

We conducted a scoping review, having registered the protocol *a priori*. A comprehensive literature search across several databases, including the grey literature, was carried out. Studies of all methodological designs were included provided they utilized, analyzed, and/or critiqued burn registry data. Pilot projects from registries in development were included as well. Studies involving non-burn specific registries or registries from a single burn centre were excluded.

**Results:**

Two hundred and sixty-eight studies were included, encompassing 16 existing burn registries. Although registry science has been used to investigate burn care since 1970, the majority of studies were published after 2007. Most studies utilized the American Burn Association Burn Registry or one of its previous versions (75.7%) and the Burns Registry of Australia and New Zealand (10.4%). Main limitations of existing registries are the inclusion of patients admitted to burn centres only, deficient capture of outpatient and long-term outcome data, and lack of data standardization across registries.

**Conclusions:**

Registries are an invaluable source of data for research, delivery of care planning, and benchmarking of processes and outcomes. Efforts should be made to stimulate other jurisdictions to build and maintain burn registries, to incorporate data linkage from administrative and other secondary databases, and to standardize data collection, in order to maximize the potential of registry science in burn care.

